# Widespread monoclonal IgE antibody convergence to an immunodominant, proanaphylactic Ara h 2 epitope in peanut allergy

**DOI:** 10.1016/j.jaci.2023.08.035

**Published:** 2024-01

**Authors:** Derek Croote, Joyce J.W. Wong, Cyprien Pecalvel, Edouard Leveque, Natacha Casanovas, Jasper B.J. Kamphuis, Paige Creeks, Johanna Romero, Saba Sohail, Daniel Bedinger, Kari C. Nadeau, Rebecca S. Chinthrajah, Laurent L. Reber, Henry B. Lowman

**Affiliations:** a IgGenix Inc, South San Francisco, Calif; b Toulouse Institute for Infectious and Inflammatory Diseases (Infinity), UMR 1291, University of Toulouse, INSERM, CNRS, Toulouse, France; c Carterra Inc, Salt Lake City, Utah; d Sean N. Parker Center for Allergy and Asthma Research, Stanford University School of Medicine, Stanford, Calif; e Department of Medicine, Stanford University School of Medicine, Stanford, Calif

**Keywords:** Human monoclonal IgE antibodies, allergy, food allergy, peanut allergy, Ara h 2, IgE, passive systemic anaphylaxis, mast cell activation tests, single-cell RNA sequencing, scRNA-Seq

## Abstract

**Background:**

Despite their central role in peanut allergy, human monoclonal IgE antibodies have eluded characterization.

**Objective:**

We sought to define the sequences, affinities, clonality, and functional properties of human monoclonal IgE antibodies in peanut allergy.

**Methods:**

We applied our single-cell RNA sequencing–based SEQ SIFTER discovery platform to samples from allergic individuals who varied by age, sex, ethnicity, and geographic location in order to understand commonalities in the human IgE response to peanut allergens. Select antibodies were then recombinantly expressed and characterized for their allergen and epitope specificity, affinity, and functional properties.

**Results:**

We found striking convergent evolution of IgE monoclonal antibodies (mAbs) from several clonal families comprising both memory B cells and plasmablasts. These antibodies bound with subnanomolar affinity to the immunodominant peanut allergen Ara h 2, specifically a linear, repetitive motif. Further characterization of these mAbs revealed their ability to single-handedly cause affinity-dependent degranulation of human mast cells and systemic anaphylaxis on peanut allergen challenge in humanized mice. Finally, we demonstrated that these mAbs, reengineered as IgGs, inhibit significant, but variable, amounts of Ara h 2– and peanut-mediated degranulation of mast cells sensitized with allergic plasma.

**Conclusions:**

Convergent evolution of IgE mAbs in peanut allergy is a common phenomenon that can reveal immunodominant epitopes on major allergenic proteins. Understanding the functional properties of these molecules is key to developing therapeutics, such as competitive IgG inhibitors, that are able to stoichiometrically outcompete endogenous IgE for allergen and thereby prevent allergic cascade in cases of accidental allergen exposure.

Peanut allergy is increasingly common, affecting an estimated 2% of children and 1.8% of adults in the United States.^1^^,^^2^ Reactions can often be severe: in the United States, 87% of food-related anaphylactic fatalities are caused by either peanut or tree nuts.^3^ Peanut is an allergen characterized by 2 immunodominant allergenic proteins: Ara h 2 and its homolog, Ara h 6, with other allergens being defined but of lesser clinical importance.^4^, ^5^, ^6^ Despite our knowledge of these allergens, which includes a crystal structure of Ara h 2,^7^ a molecular understanding of allergen recognition by monoclonal IgE antibodies has been lacking.

Assays based on plasma or sera have been useful in identifying linear IgE epitopes on these allergens^6^^,^^7^ and even building models for diagnosing allergy.^8^ However, bulk measurements such as these leave important questions unanswered regarding the sequences, affinities, clonality, and paratopes of the monoclonal IgE antibodies that mediate allergic reactivity via the cross-linking of the high-affinity IgE receptor FcεRI on the surface of mast cells and basophils.

To answer these questions, we applied our single-cell RNA sequencing IgE discovery engine^9^, ^10^, ^11^ to a diverse population of individuals with peanut allergy. Our results revealed a striking convergence of IgE monoclonal antibodies (mAbs) targeting a repetitive motif exclusive to Ara h 2. Further characterization of these mAbs revealed their high affinity and ability to single-handedly cause affinity-dependent degranulation of human mast cells and systemic anaphylaxis on peanut allergen challenge in humanized mice. Last, we demonstrated that these mAbs, reengineered as IgGs, significantly inhibit Ara h 2– and peanut-mediated mast cell degranulation.

## Methods

### IgE antibody discovery

This study was conducted in accordance with US regulations and policies governing human subject research and received institutional review board approval (no. 0000635, protocol reference Pro00049174) before subject screening and obtaining patient consent. IgE and IgG antibodies were discovered using single-cell RNA sequencing as previously described.^9^ In brief, fresh whole blood collected in either EDTA or heparin vacutainers first underwent plasma extraction by centrifugation. Plasma was stored at −80°C for subsequent allergen specific plasma IgE measurements performed with the ImmunoCAP system (Thermo Fisher Scientific). With the remaining cellular fraction, human B cells were enriched using Ficoll-based SepMate separation (STEMCELL Technologies). Remaining red blood cells were lysed, after which cells were washed and stained before single-cell sorting on the SH800 or MA900 devices (Sony Biotechnology) according to previously described gating.^9^ Single B cells were sorted into 96- or 384-well plates containing lysis buffer, centrifuged, and immediately stored at −80°C.

A modified version of the Smart-seq2 protocol^12^ was used similar to that previously described^9^ in order to prepare single B-cell transcriptomes. Sorted plates underwent reverse transcription followed by PCR to generate and amplify full-length cDNA. The cDNA was purified with a 0.8× ratio of KAPA Pure beads (Roche), normalized to approximately 0.2 ng/μL after measurement by Quant-iT PicoGreen (Thermo Fisher Scientific), and then subjected to Nextera XT (Illumina) library preparation with IDT for Illumina 10 bp dual unique indices. The Dragonfly Discovery and Mosquito HV instruments (SPT Labtech) were used for liquid handling. Libraries were pooled and sequenced on Illumina NextSeq 500 or NovaSeq instruments with 2 × 150 bp read length.

Snakemake^13^ pipelines executed on Google Cloud Platform were used to process next-generation sequencing (NGS) data for gene expression analysis and antibody assembly. STAR^14^ and ‘htseq’^15^ were used as previously described^9^ to calculate gene expression. BraCeR^16^ was used to assemble heavy and light chain sequences. Only cells with at least 500 expressed genes and a single productive pair of heavy and light chain sequences that met the following criteria were retained: heavy chain variable region (VH) and light chain variable region (VL) V gene lengths greater than 250 nt, no stop codons, no ambiguous nucleotides, correct reading frames, and unambiguous constant regions. To generate clonal families (CFs) of cells with related antibody sequences, cells were first grouped according to shared VH V gene, VH J gene, and complementarity-determining region (CDR) 3 length. Within each group, members were assigned to CFs on the basis of 70% or higher shared VH CDR3 sequence identity.

### Antibody expression and characterization

Recombinant IgE and IgG_4_ mAbs were expressed in Chinese hamster ovary (CHO) or human embryonic kidney (HEK) 293-F cells and purified by affinity chromatography. Binding of IgG_4_ antibodies at 10 mg/L to recombinant peanut allergens Ara h 1, Ara h 2, Ara h 3, Ara h 6, Ara h 8, and Ara h 9 was quantified on the ImmunoCAP system using the IgG_4_ specific conjugate and reagents. The Carterra LSA device was used to perform epitope binning of IgG_4_ mAbs as well as affinity measurements of IgG_4_ mAbs against natural Ara h 2 (InBio). Circular dichroism was used to confirm natural Ara h 2 folding relative to literature reports and folding of other 2S albumins, including natural Ara h 6 (InBio) and recombinant Ana o 3 (InBio and in-house preparation). Linear peptide array epitope mapping of select mAbs was performed using synthesized peptides derived from the Ara h 2.0201 isoform (UniProt accession no. Q6PSU2-1). A molecular model of Ara h 2.0201 (less the signal peptide sequence) was made with UCSF ChimeraX^17^ using the structure predicted with AlphaFold.^18^ A competitive luciferase-linked immunosorbent assay (LuLISA)^19^ was used to evaluate the ability of a peptide containing the repetitive motif to block binding of select IgE mAbs to Ara h 2. Further details regarding these methods and procedures are in the Methods available in this article’s Online Repository available at www.jacionline.org.

### Primary human mast cells

CD34^+^ precursor cells were isolated from peripheral blood mononuclear cells of healthy donors (provided by the French Blood Bank EFS) and cultured for ∼10 weeks, at which time >95% of all cells were CD117^+^FcεRI^+^ by flow cytometry. Further culturing details are available in the Methods in the Online Repository. Mast cells were sensitized overnight with IgE mAbs PA12P3D08, PA12P3F10, PA13P1H08, or, as a control, the anti–Ara h 6 IgE mAb 15C2 at 1 μg/mL. Cells were then washed and stimulated with 1 μg/mL of peanut extract (XPF171D3A25, Greer), 100 ng/mL of recombinant Ara h 2 (ExBio, 10-P208-C100) or 1 μg/mL of a peptide containing the repetitive motif (DPYSPSQDPYSPSQDPDRRDPYSPSPY, obtained from Thermo Fisher Scientific) in Tyrode buffer. Mast cell degranulation was measured by flow cytometry using fluorescent avidin (5 μg/mL; A2170 Invitrogen), which binds to heparin contained in mast cell granules. Data were acquired by a MACSQuant MQ10 flow cytometer (Miltenyi Biotec) and analyzed by FlowJo v10.8.1 software (Becton Dickinson).

### Animal models

The hIgE/hFcεRI mice were obtained from GenOway for passive systemic anaphylaxis experiments. All animal care and experimentation were conducted in compliance with the guidelines of the European Union (86/609/EEC) and the French Committee of Ethics (87/848) policies and with the specific approval from the local ministry-approved committee on ethics in animal experimentation (Ethics Committee UMS006 CEEA-122, project no. 2019030110022741). Mice were sensitized intraperitoneally with 10 μg of the IgE mAbs PA12P3D08, PA12P3F10, PA13P1H08, or, as a control, IgE against hapten 4-hydroxy-3-nitrophenacetyl. Sixteen hours later, mice were challenged with 1 mg of peanut extract (XPF171D3A25, Greer) to induce anaphylaxis. This peanut extract was confirmed to contain Ara h 2 by SDS-PAGE as well as Western blot analysis with IgG_4_ PA13P1H08. Anaphylaxis was monitored by measuring changes in body temperature over 1 hour. Anaphylaxis was quantified by measuring loss of core body temperature with a rectal thermometer. Temperature was measured every 10 minutes starting immediately before (time 0) and at different time points for up to 1 hour after peanut challenge, at which time sera were collected for quantification of mast cell–specific protease (MCPT) 1 and MCPT7 by ELISA. Further details are available in the Methods in the Online Repository.

### Mast cell activation assay using Hoxb8 mast cells

Mast cell activation tests (MATs) with Hoxb8 cells expressing human FcεRIα were conducted in 2 phases at ATANIS Biotech. In the first phase, the half-maximal activation concentration of 2 allergens, LoTox Peanut Flour Protein with Defined Allergen Content (InBio) and natural Ara h 2 (InBio), was determined for each peanut-allergic plasma. The second phase of the MAT assessed the inhibitory activity of IgG_4_ antibodies. Cells were sensitized with peanut-allergic plasma, washed, incubated with mAb, and stimulated with the optimal activation concentration of allergen determined in phase 1. Cellular degranulation was assessed by CD107a expression by flow cytometry, and inhibition was calculated on the basis of the degranulation of no-antibody control wells. Further details are available in the Methods in the Online Repository.

### Statistical analysis

GraphPad Prism v9.2.0 (283) (GraphPad Software) was used for statistical analysis and graphical representation. Statistical analyses were carried out by Kruskal-Wallis test, with Dunn correction for multiple comparisons or Bonferroni multiple comparison test, as indicated. Results were considered significant at *P* ≤ .05. Heat maps, cluster maps, and correlation plot matrices were generated by ‘seaborn,’^20^ and logo plots were generated by Logomaker software.^21^

## Results

### Convergent evolution of human IgE mAbs

**Fig E1:**
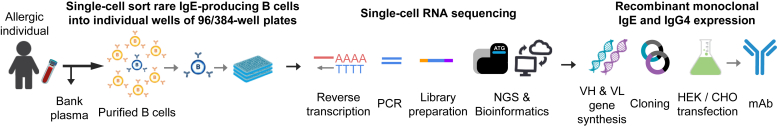
Workflow illustration. Whole blood is collected from allergic individuals and plasma is banked. The cellular fraction is processed to a single B-cell suspension, and a cell sorter is used to sort rare, IgE-producing B cells into individual wells of 96- or 384-well plates. Single-cell RNA sequencing transforms mRNA of each single B cell into a barcoded library of cDNA suitable for NGS. Full-length paired heavy and light chain sequences comprising individual mAbs are derived for each single B cell separately using cloud-based bioinformatic pipelines. Paired variable regions (VH and VL) of each mAb are cloned into heavy and light chain IgE or IgG_4_ expression vectors after gene synthesis. The expression vectors are cotransfected into HEK or CHO cells and the resulting mAb is purified from culture supernatant by affinity chromatography.

We acquired fresh whole blood samples from pediatric and adult individuals with peanut allergy. In addition to age, individuals varied in sex, ethnicity, and geographic location (Table I). An overview of our workflow, described below, is available in Fig E1 in the Online Repository available at www.jacionline.org.

**Table I:** Subject demographics

Subject ID	Age (years)	Sex	Ethnicity	Food allergies	Other atopic conditions	Site location (US state)
A	28	F	White	PN, SF, soy	Asthma	Maryland
C	16	F	Black	PN, E, pork, SF, TN	Asthma	Maryland
K	23	M	White, Hispanic	PN, E, SF, soy, TN	Eczema	California
M	12	F	Multiracial	PN, C, D, E	Asthma	Colorado
PA12	7	F	White	PN, soy, TN	Eczema	California
PA13	8	M	White, Asian	PN	None	California
Q	51	M	White	PN, TN	Asthma, eczema	Washington
V	12	F	Asian	PN, TN	Asthma, allergic rhinitis, eczema	California

PA12 and PA13 were described previously and are included here for completeness.^9^ Food allergy abbreviations are as follows: *C,* cat; *D,* dog; *E,* egg; *PN,* peanut; *SF,* shellfish; *TN,* tree nuts.

For each blood sample, we processed both plasma and cellular components. Plasma was banked for IgE titer measurements and for use in MATs. As depicted in Table E1 in the Online Repository available at www.jacionline.org, individuals varied both in total IgE and peanut-specific IgE, but all were polysensitized to multiple Ara h allergen components, with the exception of subject M, for whom overall peanut and Ara h component–specific IgE titers were low. In line with other reports of peanut-allergic individuals,^4^ the component with the highest average specific IgE (sIgE) titer was Ara h 2, while Ara h 9 and Ara h 8 titers were lowest.

We applied our SEQ SIFTER IgE discovery engine to the cellular fraction of each blood sample remaining after plasma banking. Starting with a purified, single B-cell suspension, rare IgE-producing B cells, as well as a small number of B cells producing antibodies of other isotypes, were sorted, 1 cell per well, into 96- or 384-well plates. These plates were processed through our single-cell RNA sequencing pipeline with the aid of liquid handling instrumentation; this involved, for each cell independently, lysis, mRNA reverse transcription, PCR amplification, cDNA purification, cDNA quantification, and library preparation. cDNA from each cell was molecularly barcoded with dual unique indices during this final library preparation step, which allowed for NGS reads to be assigned to their single cell of origin. Parallelized cloud-based bioinformatic pipelines were used to independently perform alignment and assembly for each single cell’s NGS data. The outputs of these steps were, for each single B cell, full transcriptomic gene expression data as well as paired heavy and light chain IgE or IgG mAb sequences.

**Fig 1:**
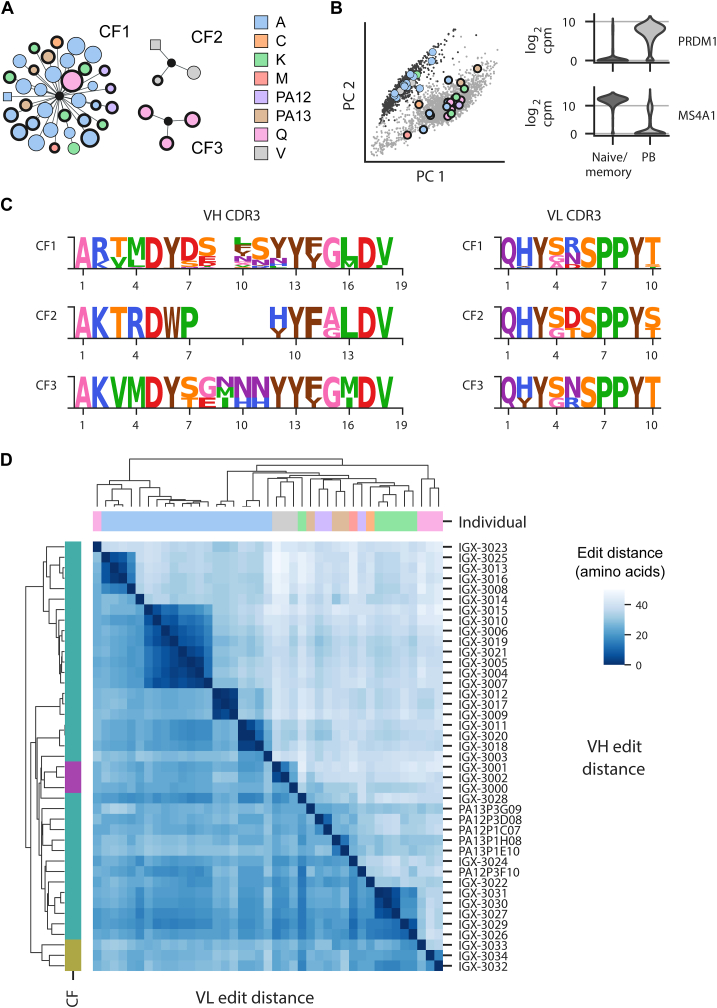
Convergent evolution and mAb sequence similarity of 3 distinct CFs of cells isolated from peanut-allergic individuals. **(A)** Network plots of CF1, CF2, and CF3. Each of the 41 points is a mAb, colored by subject of origin. *Circle* indicates IgE; *square,* IgG; *thick outline,* plasmablast; and *thin outline,* memory B cell. mAb somatic hypermutation is proportional to point size. **(B)** Cells partition by principal component (PC) analysis into plasmablast *(light)* or naive and memory *(dark)* clusters. Cells from *(A)* have been enlarged. *cpm,* Counts per million. **(C)** Aligned logo plots of CF1, CF2, and CF3 VH and VL CDR3 sequences with residues colored by functional group. **(D)** Pairwise edit distance cluster map of 41 mAbs illustrate individual-intrinsic heterogeneity. *Upper* and *lower triangles* indicate VH and VL pairwise edit distances, respectively. Columns colored by subject; rows colored by CF (*green,* CF1; *purple,* CF2; *gold,* CF3).

We obtained more than 2450 paired heavy and light chain antibody sequences, from which we built CFs of related B cells by clustering them according to VH sequence similarity. We subsequently focused on 3 CFs that stood out on the bases of sequence similarity to previously characterized IgE antibodies and a signature of convergent evolution (Fig 1, *A*). Our restricted focus on these 3 CFs, containing 41 cells, at the expense of over 2400 other cells is one limitation of this work; our findings should not be interpreted as a broad analysis of the IgE repertoires of these subjects but rather as a deep characterization of convergent antibody evolution toward a key anaphylactic epitope driving peanut allergy.

Cells from these 3 CFs partitioned into both plasmablast and memory B-cell compartments as illustrated by their respective expression of genes encoding the plasmablast transcription factor PRDM1 or the naive/memory marker gene *MS4A1* (which encodes CD20) (Fig 1, *A* and *B*), but there was a bias toward the plasmablast compartment, confirming our earlier findings on a more limited set of peanut-allergic subjects.^10^ All mAbs were IgE except for a single IgG in CF1 and a single IgG in CF2 (Fig 1, *A*).

CF1 contained previously reported antibody sequences^9^ as well as mAbs that we discovered from 5 additional individuals (Table I). CF2 contained mAbs from a single individual, but their gene usage pattern and sequences were strikingly similar to those of CF1, particularly within the light chain variable region (VL). CF3 also had highly similar gene usage, but with a VH CDR3 that was longer than that of either CF1 or CF2 (Fig 1, *C*).

Besides containing antibodies from 7 unique individuals derived from a mixture of memory B cells and plasmablasts, CF1 was unique in that it demonstrates clonal persistence within a single subject, subject A. This subject contributed the most mAbs because we performed our discovery workflow on multiple blood draws over a span of 238 days. From each of 4 consecutive draws, and then from an eighth draw, we discovered mAbs that belonged to CF1, indicating strong clonal persistence, that, according to correspondence with the allergic individual, had occurred in the absence of known allergen exposure during that period. Interestingly, we discovered memory IgE B cells at all 4 visits while discovering plasmablasts from only visits 1 and 3. This observation suggests that further research is necessary to understand apparent allergen-independent immunological memory underpinning clonal persistence of IgE-producing B cells in allergy.

Antibodies from all 3 CFs are highly similar; by edit distance, CF2 VHs are even more similar to some CF1 mAbs than some CF1 mAbs (Fig 1, *D*). Both within and between CFs, the VL CDR3 sequences were of equivalent length and highly conserved in sequence, while the ends of the VH CDR3 sequences were also largely conserved. Unsurprisingly, the center of the VH CDR3 contained the largest source of sequence diversity across CFs (Fig 1, *C*).

**Fig E2:**
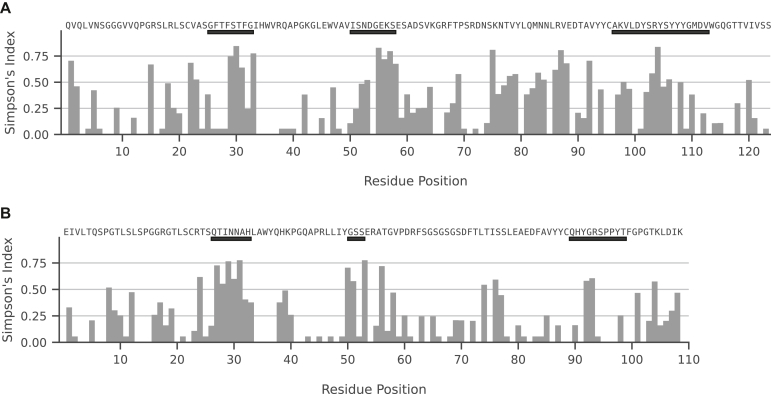
Sequence diversity in CF1. Diversity as a function of position across the VH **(A)** and VL **(B)** of CF1 as calculated by Simpson index. VH and VL sequences of PA13P1H08 are shown for reference at the top of each figure, with CDRs underlined. VH and VL sequences of PA13P1H08 are as follows: VH, QVQLVNSGGGVVQPGRSLRLSCVASGFTFSTFGIHWVRQAPGKGLEWVAVISNDGEKSESADSVKGRFTPSRDNSKNTVYLQMNNLRVEDTAVYYCAKVLDYSRYSYYYGMDVWGQGTTVIVSS; and VL, EIVLTQSPGTLSLSPGGRGTLSCRTSQTINNAHLAWYQHKPGQAPRLLIYGSSERATGVPDRFSGSGSGSDFTLTISSLEAEDFAVYYCQHYGRSPPYTFGPGTKLDIK.

Despite belonging to a CF, sequences within CF1 exhibited heterogeneity with VH edit distances as high as 40 amino acids between members. Unsurprisingly, VH and VL CDRs featured high levels of sequence diversity, although this was also true to a lesser degree of portions of the framework regions, and for the VH frameworks more than VL frameworks (see Fig E2 in the Online Repository available at www.jacionline.org). Intriguingly, evidence for individualistic affinity maturation processes that produced these mAbs can be seen in the hierarchical substructure of sequence similarity that groups mAbs by individual (Fig 1, *D*).

### mAb specificity and affinity

**Fig 2:**
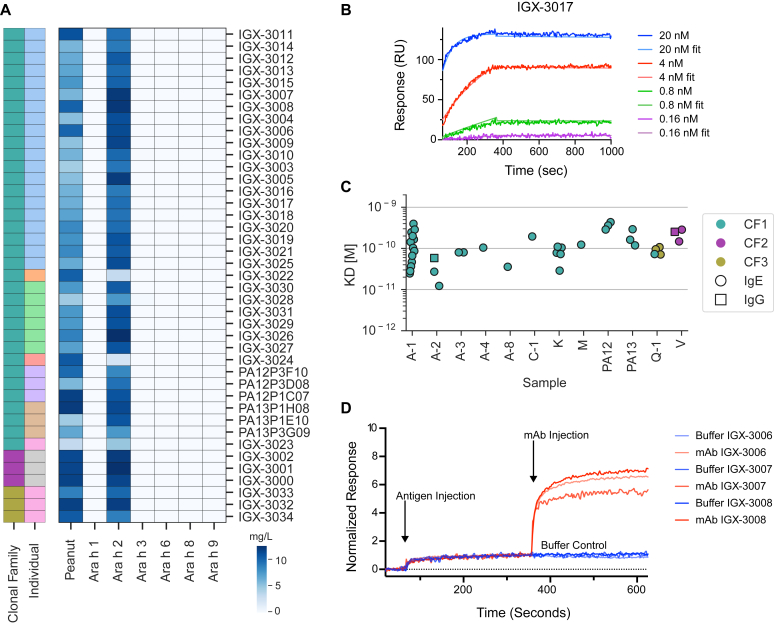
mAbs exhibit high-affinity binding to rAra h 2 and self-sandwiching behavior. **(A)** CF1, CF2, and CF3 IgG_4_ mAb binding to peanut components on ImmunoCAP. mAbs were diluted to 10 mg/L for testing. Rows (mAbs) are colored according to CF *(outer)* and individual *(inner).***(B)** Example sensorgram curves demonstrating high-affinity, sub–100 pM binding. **(C)** mAb affinity by blood sample *(column),* CF *(color),* and isotype *(shape).* Each point is a mAb. Samples with a numerical suffix correspond to a unique sample from the same subject. **(D)** Surface-bound mAb ligands are unable to block binding of themselves as analytes to captured Ara h 2 (antigen), as depicted by sensorgram curve signal increases on analyte mAb injection. Three representative mAbs are shown.

We expressed all 41 of these mAbs as IgG_4_s with the S228P stabilizing hinge mutation to avoid Fab arm exchange^22^ and characterized their specificity using the IgG_4_ detection conjugate on the ImmunoCAP system. We assessed their binding to all available peanut components: Ara h 1, Ara h 2, Ara h 3, Ara h 6, Ara h 8, and Ara h 9. Notably, all are recombinant and not naturally purified to avoid any potential cross-contamination. As depicted in Fig 2, *A,* all mAbs bound strongly and exclusively to rAra h 2.

We next assessed the affinity of these mAbs for natural Ara h 2 using the Carterra LSA. All mAbs had subnanomolar affinity, and among them, 19 (46%) had an equilibrium dissociation constant (*K*_D_) below 100 pM. Such high affinity (Fig 2, *B*) was an antibody property generally irrespective of individual of origin (Fig 2, *C*).

Epitope binning of these mAbs on the Carterra LSA produced an intriguing result. Typically, in conventional binning when pairs of antibodies from the same CF are competed in pairwise fashion, the binding of the antigen (Ara h 2) by the first antibody (ligand) inhibits the binding of the second antibody (analyte), given that their sequence similarity all but guarantees that ligand and analyte mAbs overlap in epitope. However, these mAbs did not demonstrate competition. Instead, they exhibited “universal sandwiching,” whereby ligands are unable to block analytes from binding Ara h 2. Most strikingly, this included cases of self-sandwiching—that is, an antibody, as a ligand, was unable to block the binding of itself, as an analyte, to Ara h 2 (Fig 2, *D*). In contrast, preincubating Ara h 2 and antibody before injection abrogated universal sandwiching (data not shown). Together, these data led us to hypothesize that all of these mAbs may be binding a repetitive epitope on Ara h 2.

### Linear peptide array epitope mapping

**Fig 3:**
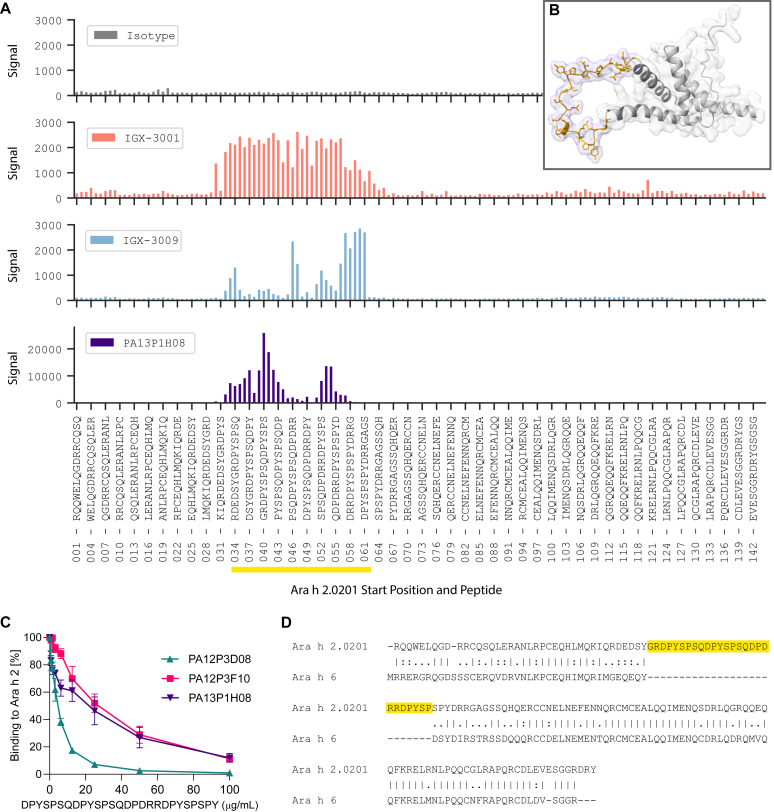
mAbs bind to a repetitive motif present on the flexible loop exclusive to Ara h 2. **(A)** Linear peptide array epitope mapping of 3 mAbs and an isotype control to Ara h 2.0201 15 mers offset by 1 amino acid. Every third peptide is labeled; peptides comprising the loop are highlighted. **(B)** Molecular model of Ara h 2.0201 highlighting residues comprising the repetitive motif *(orange).***(C)** Binding of 3 IgE mAbs from CF1 to Ara h 2 in the presence of increasing concentrations of peptide containing the repetitive motif. Data are means ± SDs pooled from 2 independent experiments. **(D)** Pairwise sequence alignment of Ara h 2.0201 with Ara h 6. Loop unique to Ara h 2 is highlighted.

We performed linear peptide array epitope mapping to determine if representative mAbs from CF1 and CF2 bound linear subsequences within Ara h 2. In line with our epitope binning results, members of both CFs bound to a repetitive motif, DPYSPS, within the flexible loop of Ara h 2 that connects large and small subunits (Fig 3, *A* and *B*).

To confirm this specificity, we found that a peptide containing the repetitive motif can block binding of select mAbs from CF1, produced as human IgE, to recombinant Ara h 2 in a dose-dependent manner (Fig 3, *C*).

As additional confirmation, we assessed whether 2 of the antibodies, IGX-3001 and IGX-3009, bound any linear epitope on Ara h 6, which shares 59% homology with Ara h 2.^23^ Neither did (data not shown), as expected because of the aforementioned absence of Ara h 6 binding (Fig 2, *A*) and the absence of the loop-containing repetitive motif within Ara h 6 (Fig 3, *D*).

### mAb proanaphylactic properties

**Fig 4:**
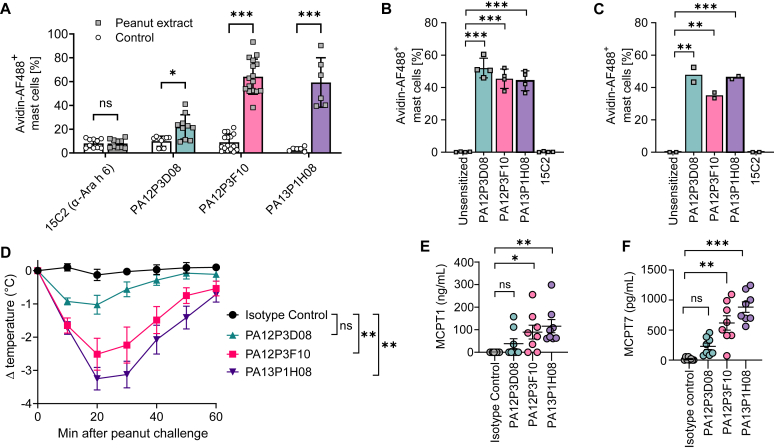
Proanaphylactic potential of monoclonal IgE targeting the repetitive Ara h 2 motif. **(A-C)** Primary human mast cells sensitized overnight with the indicated IgE mAb and stimulated with peanut extract *(A)*, recombinant Ara h 2 *(B)*, or peptide containing the repetitive DPYSPS motif *(C)*. Data show individual values, with bars indicating means ± SDs pooled from at least 3 *(A),* 4 *(B),* or 2 *(C)* independent experiments performed with mast cells derived from 3 to 6 donors. **(D)** hIgE/hFcεRI^KI^ mice sensitized intraperitoneally (i.p.) with the indicated IgE mAb and 16 hours later challenged i.p. with peanut extract. Body temperature data are indicated as means ± SEMs pooled from 2 independent experiments (n = 7-8 per group). (**E** and **F**) Levels of mast cell–specific proteases MCPT1 *(E)* and MCPT7 *(F)* measured by ELISA in serum samples collected 1 hour after peanut challenge. Data show individual values, with bars indicating means ± SEMs pooled from 2 independent experiments. *ns,* Not significant, ∗*P* < .05, ∗∗*P* < .01, ∗∗∗*P* < .001 by either Bonferroni multiple comparison test *(A-C)* or Kruskal-Wallis test *(D-F).*

We next assessed the ability of select mAbs from CF1 (PA12P3D08, PA12P3F10, PA13P1H08), produced as human IgE, to induce degranulation of human mast cells derived *in vitro* from CD34^+^ blood progenitors from healthy donors (Fig 4, *A*).^24^ Mast cells were sensitized overnight with the mAbs or with a commercially available anti–Ara h 6 (15C2) IgE as control. Cells were then stimulated with whole peanut extract, and mast cell degranulation was assessed by flow cytometry that used a fluorescent avidin probe (which binds heparin contained in mast cell secretory granules).^25^ We observed strong degranulation in cells sensitized with PA12P3F10 and PA13P1H08, and to a lesser extent PA12P3D08 (Fig 4, *A*), which reflects the higher affinity of PA13P1H08 and PA12P3F10 relative to PA12P3D08. In contrast, no degranulation was observed with the anti–Ara h 6 mAb (Fig 4, *A*). In line with these results, the 3 CF1 IgE mAbs, but not the anti–Ara h 6 IgE mAb, induced mast cell degranulation on stimulation with recombinant Ara h 2 or a peptide containing the repetitive motif (Fig 4, *B* and *C*). All together, these results highlight that targeting the repetitive motif within the flexible loop of Ara h 2 is key to induce cross-linking of FcεRI and subsequent mast cell degranulation.

To further confirm the anaphylactic potential of these mAbs, we performed passive systemic anaphylaxis experiments in hIgE/hFcεRI transgenic mice,^26^ which express human FcεRIα and human IgE heavy chain in place of the respective mouse proteins. Mice were sensitized with a single injection of 1 of 3 IgE mAbs targeting the repeated Ara h 2 epitope (PA12P3D08, PA12P3F10, PA13P1H08) or an isotype control and challenged 1 day later with peanut extract to induce systemic anaphylaxis. As a readout of anaphylaxis, we measured changes in body core temperature.^27^ In line with our data in human mast cells, we found that these mAbs were able to induce systemic anaphylaxis; we also found that the severity of the anaphylactic shock correlated with the respective affinity, in rank order (highest affinity to lowest: PA13P1H08, PA12P3F10, PA12P3D08), of the 3 mAbs (Fig 4, *D*). We also found elevated levels of the mast cell–specific chymase MCPT1 (Fig 4, *E*) and tryptase MCPT7 (Fig 4, *F*) 1 hour after challenge in serum samples from mice sensitized with PA13P1H08, PA12P3F10, and, to a lesser extent, PA12P3D08, confirming the ability of these mAbs to induce degranulation of both mucosal and connective tissue–type mast cells *in vivo*.

While antibodies in the plasma from allergic individuals are polyclonal and thereby confounded by unknown IgE diversity and mAb affinity, our data obtained using IgE/FcεRI-humanized mice and monoclonal, monospecific IgE mAbs provide a simplified system to understand the functional relevancy of a singular epitope. The ability of individual IgE mAbs to elicit anaphylaxis in an affinity-dependent manner solidifies the clinical importance of this previously identified epitope.^23^^,^^28^ It also provides direct *in vivo* evidence for one mode of effector cell cross-linking: that IgE on the same effector cell with the same specificity can bind independent copies of the same repeated Ara h 2 epitope.^29^

### Ability of reengineered mAbs to inhibit mast cell degranulation

Having established the anaphylactic potential of these mAbs as IgE, we evaluated the ability of selected mAbs, reengineered as IgG_4_ mAbs, to inhibit Ara h 2– and peanut-mediated degranulation using the recently described Hoxb8 mast cell line.^30^ Cells were sensitized with plasma from peanut-allergic subjects to capture polyclonal human IgE, incubated with IgG_4_ antibody, and then stimulated with Ara h 2 or peanut flour protein to induce degranulation. Notably, this peanut flour protein contained measured quantities of Ara h 1, 2, 3, and 6 (see the Methods section in the Online Repository). Mast cell activation was quantified using flow cytometry by measuring surface levels of the lysosome-associated membrane protein-1/CD107a that get exposed during degranulation.^30^ In order to achieve sufficient dynamic range in degranulation necessary to accurately quantify inhibition, the concentration of peanut flour protein and natural Ara h 2 necessary for half-maximal degranulation was first established for each of 20 plasmas, which varied in total IgE, peanut sIgE, and peanut-component sIgE titers (see Table E2 in the Online Repository available at www.jacionline.org).

**Fig 5:**
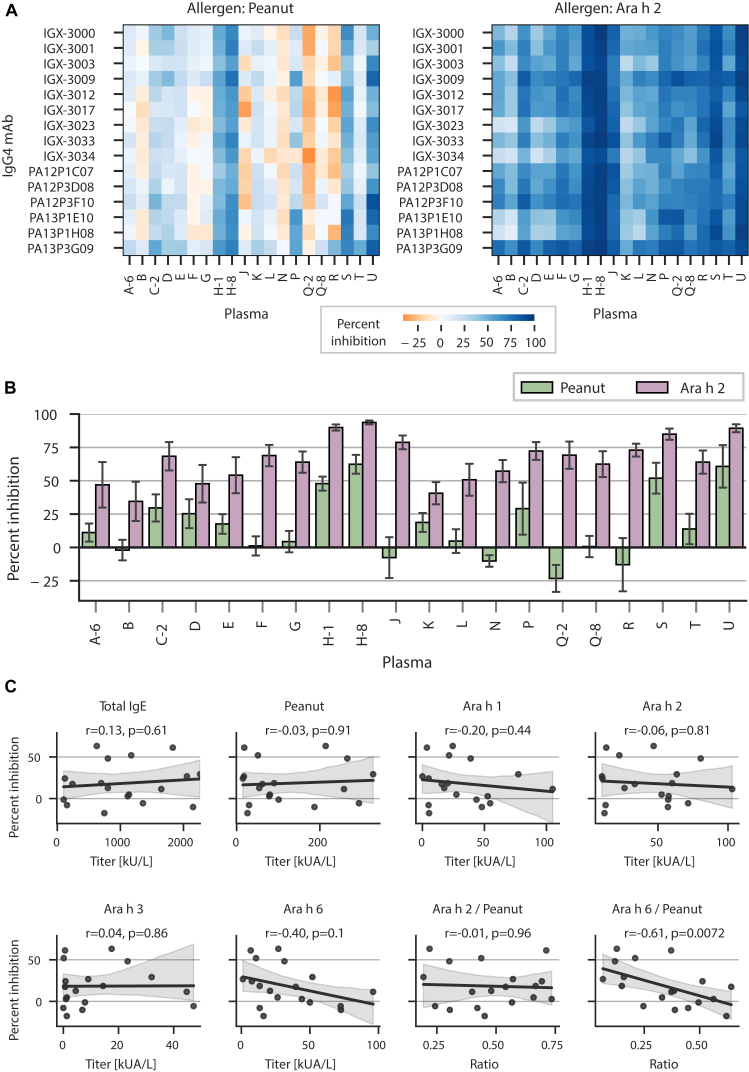
Inhibition of mast cell degranulation by IgG_4_ mAbs. **(A)** Heat map depicting inhibition of peanut- *(left)* or Ara h 2– *(right)* mediated mast cell degranulation by IgG_4_ mAbs as function of plasma. Plasmas with a numerical suffix indicate a separate blood sample from the same subject (Tables E1 and E2). **(B)** Aggregate inhibition by plasma and allergen. Data are indicated as means ± SDs of all IgG_4_ mAbs depicted in *(A).***(C)** Correlation of IgE titers or ratios thereof with median peanut percentage inhibition. Each point is a plasma sample, with Q-2 and Q-8 excluded as outliers (Table E2). Spearman correlation coefficients and associated *P* values are shown.

Next, using a predetermined allergen concentration slightly above that eliciting half-maximal activation, we assessed whether 15 mAbs, expressed as IgG_4_, could individually inhibit mast cell degranulation if added before allergen stimulation. Compared to the isotype control, mAbs significantly and consistently inhibited Ara h 2–mediated degranulation, with an average inhibition of 65% across the 20 plasmas (Fig 5, *A*). On the one hand, this strongly suggests that in all these unrelated subjects, sIgE targeting the immunodominant repetitive DPYSPS motif are driving Ara h 2–induced degranulation, which can be blocked by single reengineered monoclonal IgG_4_. On the other hand, mAbs were less able to inhibit degranulation mediated by peanut flour protein (Fig 5, *A*). Interestingly, the variability in inhibition was driven by plasma more than mAb: some plasmas were not measurably inhibited by any mAb when stimulated with peanut, while for 3 plasmas, mAbs on average inhibited more than 50% of peanut-mediated degranulation (Fig 5, *B*). All together, this indicates that in some patients, Ara h 2 is the main peanut component driving IgE reactivity, while in others, sIgE to additional peanut components or epitopes significantly contributes to peanut reactivity. We found no correlation between total IgE, peanut sIgE, or peanut-component sIgE plasma titers and percentage inhibition of peanut-mediated degranulation (Fig 5, *C*). However, the ratio of Ara h 6–specific IgE to peanut-specific IgE did significantly negatively correlate (Fig 5, *C*), supporting the contribution of Ara h 2’s complement, Ara h 6, in mediating allergic reactivity, as has been described.^4^

## Discussion

The development of new technologies that enable more sensitive measurements in immunology, such as single-cell RNA sequencing, can disrupt established beliefs, such as that convergent evolution should be, and is, a rare phenomenon given the vast possible diversity of independently recombined and paired heavy and light chain variable region sequences.^29^ However, in the case of peanut allergy, we extend a previous report of convergent evolution^9^ and demonstrate a related but distinct form of convergence to an Ara h 2 epitope by distinct CFs through slightly different heavy chain variable region recombinations.

In one subject, subject Q, we discovered IgE plasmablasts within both CF1 and CF3 (Fig 1). The high sequence dissimilarity between members suggests distinct ancestral clones affinity matured and class switched against this immunodominant epitope. Because all 4 of these IgE mAbs originated from the same blood collection, it is unknown whether such intrasubject convergence has a temporal component, but a possible line of future research could involve repeated sampling of young individuals at risk of developing an allergy to understand the contemporaneous emergence of an allergy with IgE CFs. Tracking the properties, such as somatic hypermutation and affinities, of these CFs over time has the potential to shed light on the poorly understood molecular progression of allergy.

Conventional allergy tests quantify titers of allergen-specific IgE regardless of their clonality and affinity, which may contribute to the overestimation of true clinical allergy and the poor correlation with the severity of allergic reactions. Thus, a better knowledge of the properties and convergence of IgE CFs could also lead to the design of more robust allergy diagnostic tools.

Despite the identification and naming of over a dozen peanut allergens to date, we found that a single monoclonal IgE antibody targeting a repetitive motif in Ara h 2 can elicit mast cell degranulation and anaphylaxis. Importantly, this repetitive DPYSPS motif was previously identified as an immunodominant epitope using plasma samples from patients living both in the United States and in Europe, highlighting that convergence toward this key epitope is a highly conserved mechanism in peanut allergy.^23^^,^^28^^,^^31^ In strong support of our data, it was recently demonstrated that individuals with sustained clinical tolerance induced by peanut oral immunotherapy produce IgG antibodies mainly targeting this immunodominant Ara h 2 DPYSPS motif.^32^ When aligned with our data, this strongly suggests that the efficacy of oral immunotherapy can be attributed, at least in part, to generation of key IgG mAbs competing with IgE for immunodominant epitopes. By extension, recombinant IgG neutralizing such immunodominant epitopes could be used for the treatment of allergies. Indeed, our data demonstrate that when produced as IgG_4_, single mAbs targeting the key Ara h 2 repetitive epitope can significantly inhibit degranulation of mast cells sensitized with polyclonal IgE from unrelated peanut-allergic individuals. While these data indicate that a single IgG_4_ mAb may not be adequate to sufficiently block peanut-mediated cellular degranulation across the peanut-allergic population, they nonetheless highlight the great therapeutic potential of such reengineered mAbs.

## Disclosure statement

J.B.J.K acknowledges support from the French 10.13039/501100009187Medical Research Foundation (FRM) (SPF202005011962). L.L.R acknowledges support from the INSERM, the French Medical Research Foundation (FRM EQU202103012566), the 10.13039/501100001665French National Research Agency (ANR-20-CE15-0026), and the 10.13039/501100000781European Research Council (ERC-2021-CoG 101043749).

Disclosure of potential conflict of interest: D. Croote, J. J. W. Wong, P. Creeks, J. Romero, S. Sohail., K. C. Nadeau, and H. B. Lowman are employees of and/or stakeholders in IgGenix Inc. K. C. Nadeau has received grants from the 10.13039/100000060National Institute of Allergy and Infectious Diseases (NIAID), the National Heart, Lung, and Blood Institute, the 10.13039/100000066National Institute of Environmental Health Sciences, and Food Allergy Research and Education (FARE); has an equity stake in Alladapt Immunotherapeutics, Before Brands, ClostraBio, COUR Pharmaceuticals, ImmuneID, Latitude Food Allergy Care, and Seed Health; and has received consulting fees from Excellergy Therapeutics, Red Tree Venture Capital, and Regeneron. R. S. Chinthrajah has received grants from NIAID, the 10.13039/100000002National Institutes of Health Consortium of Food Allergy Researchers, the 10.13039/100015521Stanford Maternal and Child Health Research Institute, and FARE; and has served in an advisory capacity to Alladapt Immunotherapeutics, Novartis, Genentech, Allergenis, Intrommune Therapeutics, and IgGenix. D. Bedinger is an employee of and stakeholder in Carterra Inc. L. L. Reber has received grants and/or consulting fees from Novartis, Argenx, and NEOVACS outside the submitted work. The rest of the authors declare that they have no relevant conflicts of interest.

**Clinical implications**

Human monoclonal IgE discovery identifies immunodominant allergens and epitopes that drive allergic reactivity and provides a foundation for the development of allergen-specific therapeutics capable of inhibiting IgE engagement with allergen.

## Figures and Tables

**Table E1:** Plasma IgE titers of discovery samples

Subject ID	Total	Peanut	Ara h 1	Ara h 2	Ara h 3	Ara h 6	Ara h 8	Ara h 9
A-1	1234	97.6	53.3	63.7	30.9	44.2	<0.1	<0.1
A-2	1807	486	195	192	58.8	124	<0.1	0.13
A-3	1688	692	342	353	50.4	87.7	<0.1	0.12
A-4	1315	382	141	160	39.1	97.4	<0.1	0.11
A-8	1718	432	176	185	59.7	101	<0.1	0.11
C-1	2365	261	94.1	88.8	37.6	60.2	0.12	0.15
K	697	90.1	20.5	60.1	14.4	13.4	<0.1	<0.1
M	1008	1.29	0.13	0.14	0.19	<0.1	0.39	0.3
PA12	328	154	41.1	72.7	4.6	ND	<0.1	<0.1
PA13	293	63.3	7.75	63.7	0.6	ND	17.5	0.4
Q-1	9548	80.5	90.2	69.4	30.3	70.6	40.2	0.58
V	571	251	97.3	170	9.97	69.1	<0.1	<0.1

Titers were measured by ImmunoCAP. Units are either kU/L (total IgE) or kUA/L (all others). *ND,* Not determined.

**Table E2:** IgE titers of plasmas included in Hoxb8 mast cell inhibition experiments

Subject ID	Total	Peanut	Ara h 1	Ara h 2	Ara h 3	Ara h 6	Ara h 8	Ara h 9
A-6	1649	258	105	103	44.7	96.1	<0.1	0.12
B	1358	294	54.6	71.5	47.2	72.4	8.79	0.17
C-2	2264	328	77.3	63.4	32.0	51.8	<0.1	<0.1
D	120	15.0	5.04	10.4	0.77	7.69	<0.1	<0.1
E	241	61.8	15.6	33.4	0.64	39.7	<0.1	<0.1
F	101	101	43.6	57.3	7.84	50.0	1.14	14.9
G	1116	78.4	52.6	57.6	0.95	44.1	<0.1	<0.1
H-1	802	266	38.9	80.5	23.3	31.4	<0.1	0.13
H-8	626	214	24.4	46.9	17.5	27.0	<0.1	<0.1
J	154	29.9	4.95	12.6	<0.1	13.4	<0.1	<0.1
K	697	90.1	20.5	60.1	14.4	13.4	<0.1	<0.1
L	1136	79.9	27.4	52.6	1.25	26.4	<0.1	<0.1
N	2152	187	47.9	57.1	6.90	72.6	22.3	6.13
P	2055	17.5	<0.1	10.0	9.02	1.11	<0.1	<0.1
Q-2	25040	61.7	58.7	29.3	13.7	38.9	18.2	0.59
Q-8	16741	896	538	77.4	28.6	81.5	31.6	1.40
R	746	26.1	5.38	11.8	1.25	16.2	11.8	3.35
S	1172	51.2	21.5	22.4	0.14	10.7	12.0	<0.1
T	802	54.4	17.6	26.1	4.50	20.9	3.27	<0.1
U	1831	18.4	3.63	13.1	0.61	6.83	<0.1	<0.1

Titers were measured by ImmunoCAP. Units are either kU/L (total IgE) or kUA/L (all others).

## Methods

### IgG_4_ antibody expression

GenScript’s High-throughput Gene to Antibody service was used to produce human IgG_4_ mAbs containing the S228P mutation that prevents Fab arm exchange. In brief, heavy and light chain variable region genes from each mAb were synthesized after codon optimization, cloned into pcDNA3.4 vectors containing the appropriate constant region (IgG_4_ S228P, and either λ or κ, as appropriate), and transiently cotransfected into CHO cells (HD CHO-S cell line, GenScript) maintained in ∼4-10 mL cultures for approximately 7 days. Antibodies were purified from the supernatant with 1-step protein A purification using magnetic beads. After washing and elution, the eluted fractions were pooled and buffer exchanged to the final formulation buffer: PBS, pH 7.2. Antibodies were characterized by SDS-PAGE under reducing and nonreducing conditions.

### IgE antibody expression

Anti–Ara h 2 IgE mAbs PA12P3D08 (#Ab01493-14.0) and PA12P3F10 (#Ab01494-14.0) were obtained from Absolute Antibody and the anti–Ara h 6 IgE mAb 15C2 (#E-15C2) was obtained from InBio.

Anti–Ara h 2 PA13P1H08 VH and VL sequences were reverse transcribed into DNA and codon optimized for expression in human cells using IMGT-V-Quest software. VH and VL DNA fragments were synthesized by Eurofins. The VH sequence was cloned into a human pUC19-Igε-expressing vector (the kind gift of Hugo Mouquet and Pierre Bruhns, Institut Pasteur, Paris) using *Sal*I and *Age*I restriction sites, and the VL sequence was cloned into human Igκ-expressing vector using *Age*I and *Bsi*WI restriction sites, as previously described.^E1^ Plasmids were sequenced before being used for antibody production. Antibodies were produced by transient cotransfection of PA13P1H08 IgE VH and VL expression plasmids into exponentially growing Freestyle HEK 293-F cells (Thermo Fisher Scientific) that were cultured in serum-free Freestyle 293 Expression Medium (Life Technologies) in suspension at 37°C in humidified 8% CO_2_ incubator on a shaker platform rotating at 110 rpm. Cells were cultured for 6 days after transfection. Supernatants containing IgE were collected, centrifuged at 4200 rpm for 30 minutes, and filtered (0.2 μm).

JW8/5/13 (ECACC 87080706) cells were obtained from Sigma-Aldrich. This cell line produces a chimeric human IgE antibody directed against the hapten 4-hydroxy-3-nitrophenacetyl (NP) and is composed of human Fcε chain and mouse anti-NP variable chain. JW8/5/13 cells were cultured in complete Dulbecco modified Eagle medium (Gibco) containing 2 mmol glutamine (Thermo Fisher Scientific) and 10% fetal bovine serum (Thermo Fisher Scientific) at 9 × 10^5^ cells/mL. After 15 days, supernatants were collected, centrifuged at 4200 rpm for 30 minutes, and filtered (0.2 μm).

We purified anti-NP IgE and the anti–Ara h 2 IgE PA13P1H08 by affinity chromatography using the Äkta pure system (GE Healthcare) with an XK16/20 column (28-9889-37; Cytiva) containing cyanogen bromide–activated Sepharose 4B (17-0430-01; Cytiva) coupled with the anti-IgE mAb omalizumab, as described previously.^E1^ Elution was made with glycine buffer pH 2.7 in isocratic mode. After purification, IgE antibodies were desalted by dialysis in PBS using Slide-A-Lyser Dialysis Cassette G2 (87719, Thermo Fisher Scientific), then stored at 4°C until use.

### Binding kinetics

Kinetic parameters of antibody binding to Ara h 2 were measured with a Carterra LSA instrument. Antibodies were coupled to HC30M sensor chips using 25 mmol 2-(*N*-morpholino)ethanesulfonic acid (MES), pH 5.5 (Carterra), 0.05% Tween 20 (Carterra), running buffer with the Carterra LSA multichannel printhead. Sensor chips were activated with 133 mmol 1-ethyl-3-(3-dimethylaminopropyl) carbodiimide hydrochloride (Pierce Premium Grade), 33 mmol *N*-hydroxysulfosuccinimide (aka sulfo-NHS; Pierce Premium Grade), and 33 mmol MES pH 5.5 (Carterra) buffer for 7 minutes. Antibodies were coupled to the activated surface at 0.1, 1.0, and 10.0 μg/mL concentrations in 10 mmol sodium acetate pH 4.75 (Carterra), 0.05% Tween 20 (Carterra), for 10 minutes. Remaining reactive groups were inactivated with 1 mol ethanolamine, pH 8.5 (Carterra), for 7 minutes.

Natural Ara h 2 (nAra h 2; InBio) was serially injected at 0.16, 0.8, 4, and 20 nmol concentrations using the Carterra LSA single flow cell over the antibody-coupled chip in HEPES-buffered saline–Tween 20–EDTA (HBS-TE), 0.05% bovine serum albumin (BSA) running buffer (Carterra), without regeneration between injections. Five minutes’ association time and 20 minutes’ dissociation time were used for each injection. Association and dissociation curves were fit using a 1:1 Langmuir binding model to obtain *k*_d_, *k*_a_, and *K*_D_ with Kinetics software (Carterra).

### Epitope binning

For both classical and premixed binning, surfaces were functionalized with antibodies as described above, and HBS-TE + 0.05% BSA was used as the running buffer and diluent for all proteins. For classical binning, each binding cycle consisted of 100 nmol of nAra h 2 (InBio) injected using the Carterra LSA single flow cell with 4 minutes’ association time, followed by 30 μg/mL of antibody with 4 minutes’ association time, and regeneration with two 10-second injections of 0.65% phosphoric acid (Carterra). For premixed binning, 50 μg/mL of antibody was mixed with nAra h 2 (InBio) at a 10-fold molar excess, then injected using the Carterra LSA single flow cell with 4 minutes’ association time. Analysis of epitope binning was performed by Epitope (Carterra). Binding of antibody was normalized to signal from the buffer-only injections that follow antigen injections for classical binning. Binding of antibody–antigen complex was normalized to antigen-only injections for premixed binning. Antibody pairs were classified as blocking or sandwiching according to whether they met binding signal thresholds above the buffer injection background.

### Epitope mapping

The target sequence of Ara h 2.0201 (UniProt accession no. Q6PSU2-1), the long isoform of Ara h 2, was translated into linear 15 amino acid peptides with a peptide–peptide overlap of 14 amino acids. The final several peptides were padded with serine–glycine.

The peptide array was synthesized using Fmoc-based solid-phase peptide synthesis. To verify the quality of the synthesized peptides, a separate set of positive and negative control peptides was synthesized in parallel and screened with commercial antibodies (data not shown).

Peptide binding was assessed in 1 of 2 ways. An ELISA-based approach was used for antibodies IGX-3001, IGX-3009, and an isotype control antibody. The peptide arrays were first incubated with primary antibody (overnight at 4°C). After washing, the peptide arrays were incubated with a 1/10000 dilution of a secondary antibody peroxidase conjugate for 1 hour at 25°C. After washing, ABTS and 20 μL/mL of 3% H_2_O_2_ were added. After 1 hour, color development was quantified with a charge-coupled device camera and image processing system.

Peptide binding of antibody PA13P1H08 was assessed by fluorescence. The microarray was first subjected to 15 minutes’ preswelling in washing buffer (PBS, pH 7.4 with 0.05% Tween 20), followed by 30 minutes in blocking buffer (Rockland blocking buffer MB-070). The microarray was incubated with the primary antibody at a concentration of 1 μg/mL in incubation buffer (washing buffer with 10% blocking buffer) for 16 hours at 4°C with shaking at 140 rpm. The microarray was then stained with secondary goat anti-human IgG (H+L) DyLight680 antibody (1:5000) and control mouse monoclonal anti-HA (12CA5) DyLight800 antibody (1:2000) for 45 minutes in incubation buffer at room temperature (RT). Readout was performed with the LI-COR Odyssey Imaging System with the following parameters: scanning offset 0.65 mm, resolution 21 μm, scanning intensities 7/7 (red, 700 nm; green, 800 nm). Quantification of spot intensities and peptide annotation was done with PepSlide Analyzer.

### Competitive IgE LuLISA

LuLISA^E2^ was used to evaluate the ability of a peptide encoding the repetitive motif to block binding of select IgE mAbs to Ara h 2. Briefly, white 96-well plates with flat bottoms (FluoroNunc C96 MaxiSorp, Nunc) were coated with 5 μg/mL recombinant Ara h 2 (ExBio, 10-P208-C100) in NaHCO_3_ 50 mmol buffer, 50 μL per well, for 2 hours at RT, then blocked (100 μL per well, PBS 0.1% Tween 20, 1% BSA) for 2 hours. Purified Ara h 2–specific IgE mAbs (30 ng/mL) were preincubated with the repetitive peptide (DPYSPSQDPYSPSQDPDRRDPYSPSPY, obtained from Thermo Fisher Scientific, 0.78-100 μg/mL) for 30 minutes at RT and then incubated in Ara h 2–coated plates for 1 hour. Purified sdAb026-nanoKAZ (an anti-IgE nanobody that recognizes the constant Cε3 region of human IgE and that is expressed in tandem with the catalytic domain of the enzyme luciferase) was added (1 ng/mL, 50 μL per well) and incubated for 30 minutes at RT. Plates were washed with PBS, and 50 μL of furimazin (imidazo[1,2-a]pyrazin-3(7*H*)-ones) was injected at a final concentration of 27 μmol using a plate luminometer (LB960 Centro, Berthold). The plate was orbitally shaken for 5 seconds, and the collected photons were counted per well during 1 second and measured 3 times. The bioluminescent signal obtained with each IgE mAb without incubation with the repetitive peptide was set at 100% binding.

### Primary human mast cell culture

CD34^+^ precursor cells were isolated from peripheral blood mononuclear cells of healthy donors (provided by the French Blood Bank EFS). CD34^+^ cells were maintained for 1 week under serum-free conditions using StemSpan medium (9655 STEMCELL Technologies) supplemented with recombinant human IL-6 (50 ng/mL; 200-06 PeproTech), human IL-3 (10 ng/mL; 200-03 PeproTech), and 3% supernatant of CHO transfectants secreting murine stem cell factor (SCF; the kind gift of P. Dubreuil, Marseille, France; 3% [corresponding to ∼50 ng/mL SCF]) ciprofloxacin (10 ng/mL; 17850-5G-F Sigma-Aldrich). Thereafter, the cells were maintained in IMDM GlutaMAX I (31980048), 2-mercaptoethanol (31-350-010), insulin–transferrin selenium (2506865) (all from Gibco), sodium pyruvate (S8636 Sigma-Aldrich), 0.5% BSA (A2153 Sigma-Aldrich), penicillin/streptomycin (100 U/mL/100 μg/mL; Life Technologies), IL-6 (50 ng/mL; 200-06 PeproTech), and 3% supernatant of CHO transfectants secreting mouse SCF. Before their use in experiments, mast cells were tested for phenotype by flow cytometry (CD117, FcεRI). Cells were ready for experiments after ∼10 weeks in culture (at which time >95% of all cells were CD117^+^FcεRI^+^).

### MCPT1 and MCPT7 ELISAs

Serum samples from hIgE/hFcεRI mice were collected 1 hour after peanut challenge, and concentrations of the mast cell–specific proteases MCPT1 and MCPT7 were quantified by ELISA. MCPT1 concentration was measured according to the manufacturer’s instructions (Thermo Fisher Scientific; 88-7503-88), while MCPT7 level was evaluated using an in-house sandwich ELISA. Briefly, plates were coated with anti-mouse MCPT7 antibodies (0.8 μg/mL final; R&D Systems; AF1937) overnight at 4°C. Plates were washed and blocked with a solution of PBS 0.1% Tween 20 1% BSA at RT for 1 hour. Samples and recombinant MCPT7, used as a standard (R&D Systems; 1937-SE-020), were incubated for 2 hours at RT. Plates were then washed and incubated for 1 hour at RT with a biotin-conjugated anti-mouse MCPT7 antibody (0.8 μg/mL final; R&D Systems; BAF1937). After washing, streptavidin-HRP (4 μg/mL; R&D Systems; DY998) was added for 30 minutes at RT. Plates were washed before addition of 3,3′,5,5′-tetramethylbenzidine (aka TMB) solution. The reaction was stopped by addition of H_2_SO_4_, and absorbance was recorded at 450 nm.

### Mast cell activation assay using Hoxb8 mast cells

MATs with Hoxb8 cells expressing human FcεRIα were conducted in 2 phases at ATANIS Biotech. In the first phase, the half-maximal activation concentration of 2 allergens was determined for each plasma. Cells were first sensitized with patient plasma, washed, and resuspended in a half volume of medium and incubated for 30 minutes. Next, to stimulate, cells were incubated for 30 minutes with 0 to 100 ng/mL, added at 2× final concentration, of LoTox Peanut Flour Protein with Defined Allergen Content (InBio) or natural Ara h 2 (InBio). The lot of peanut flour protein used contained the following allergen concentrations according to the certificate of analysis: 226 μg/mL Ara h 1, 363 μg/mL Ara h 2, 480 μg/mL Ara h 3, 168 μg/mL Ara h 6, and 5 ng/mL Ara h 8. Cellular degranulation was assessed by CD107a expression by flow cytometry, and the half-maximal activation concentration was determined from dose–response curves by GraphPad Prism.

The second phase of the MATs assessed the inhibitory activity of IgG_4_ antibodies. As in phase 1, cells were first sensitized with patient plasma and washed. Subsequently, cells were incubated with antibody for 30 minutes at a final concentration of 10 μg/mL. Without washing, the optimal activation concentration of allergen determined in phase 1 was added at 2× final concentration and incubated for 30 minutes. Stimulation was assessed as in phase 1 and inhibition calculated on the basis of degranulation of no-antibody control wells.
